# Serologic Surveillance for West Nile Virus in Dogs, Africa

**DOI:** 10.3201/eid2008.130691

**Published:** 2014-08

**Authors:** Bernard Davoust, Isabelle Leparc-Goffart, Jean-Paul Demoncheaux, Raphaël Tine, Mamadou Diarra, Grégory Trombini, Oleg Mediannikov, Jean-Lou Marié

**Affiliations:** Groupe de Travail en Épidémiologie Animale du Service de Santé des Armées, Toulon, France (B. Davoust, J.-P. Demoncheaux, G. Trombini, J.L. Marié);; Unité de Recherche sur les Maladies Infectieuses et Tropicales Émergentes (IRD 198), Dakar, Senegal (B. Davoust, O. Mediannikov);; Centre National de Référence des Arbovirus–Institut de Recherche Biomédicale des Armées, Marseille, France (I. Leparc-Goffart);; Services Vétérinaires de la Gendarmerie Nationale, Dakar (R. Tine, M. Diarra)

**Keywords:** West Nile virus, WNV, dogs, serology, Western blot, Africa, sentinel animal, France, viruses, surveillance, military working dogs, seroconversion, Europe, Hungary, Flavivirus, flaviviruses

**To the Editor:** West Nile fever is caused by the West Nile virus (WNV), a mosquito-borne member of the genus *Flavivirus*. Birds are the natural reservoir of the virus, which is maintained in nature in a mosquito–bird–mosquito transmission cycle. WNV has been detected in many regions worldwide, including North America, Europe, Africa, the Near East, and Asia (*1*). WNV has been shown to cause meningoencephalitis in humans and horses. In the United States, seroconversion in dogs was detected 6 weeks before a human case was reported (*2*). Thus, dogs could be considered as sentinels for WNV infection, but their role as reservoir is unlikely because of short-term and low levels of viremia (*3*). In this study, we determined the seroprevalence of WNV in dogs living close to humans in different environments to assess their role as sentinels of this potentially severe zoonosis.

During 2003–2012, blood samples were collected from 753 adult dogs from France and 6 countries in Africa (Table). Samples were centrifuged within 24 h after collection, separated, frozen at –20°C, and sent to the virology laboratory of the Institut de Recherche Biomédicale des Armées (Marseille, France). Each sample was systematically tested for IgG against WNV by using an in-house ELISA with inactivated WNV as antigen. Serum samples were considered positive if the optical density at 450 nm was >3-fold the mean of that for negative antigen. Because of the antigenic cross-reactivity among flaviviruses, all positive samples were further tested by Western blot for WNV-specific antibodies (*4*); seroprevalence was calculated on the basis of Western blot–confirmed cases only. For the statistical analysis, we used the exact binomial method to calculate 95% CIs of the proportions and the Fisher exact test to calculate p values and compare the seroprevalence rates between countries; significance was set at p<0.05.

**Table:** Prevalence of West Nile virus antibodies in dog populations, France and Africa, 2003–2012

Country and area	No. dogs, N = 753	No. positive for IgG by ELISA	No. results confirmed by Western blot	Prevalence, % (95% CI)
France				
Corsica	35*	3	0	0 (0–10)
Var	25*	3	3	12.0 (2.5–31.2)
Gard	11*	1	1	9.1 (0.2–41.3)
Imported from				
Germany/the Netherlands	9*	0	0	0 (0–33.6)
Hungary	24*	6	3	12.5 (2.7–32.4)
Djibouti	47*	8	6	12.8 (4.8–25.7)
N'Djamena, Chad	50*	13	12	24.0 (13.1–38.2)
	5	5	5	100.0 (47.8–100.0)
Senegal				
Dakar	11*	0	0	0 (0–28.5)
	16†	3	3	18.7 (4.1–45.6)
Siné-Saloum	33	6	1	3.0 (0.1–15.8)
Casamance	81	3	3	3.7 (0.8–10.4)
Abidjan, Côte d’Ivoire	137	7	3	2.2 (0.5–6.3)
Kinshasa, Democratic Republic of the Congo	24	4	3	12.5 (2.7–32.4)
Haut-Ogooué, Gabon	245	0	0	0 (0–1.5)

*French military working dogs. †Senegalese gendarmerie working dogs.

Seropositive dogs were found in all portions of Africa and France surveyed except northeastern Gabon and Corsica (Table). Seroprevalence of WNV in native dogs was significantly higher in Chad than in the Democratic Republic of the Congo (DRC) (p<0.001), Senegal (p<0.00001), Côte d’Ivoire (p<0.000001), and Gabon (p<0.000001). Seroprevalence was low in Kinshasa, DRC (12.5%), and Dakar, Senegal (11.1%), but in N’Djamena, Chad, all 5 native dogs tested had specific antibodies against WNV. 

As part of the study, we tested 50 military dogs from France twice, before and after a 4-month mission in Chad; 12 (24.0%) became seropositive after the stay. In addition, 12.5% of military working dogs in France imported from Hungary were seropositive on initial testing. We also found that, in France, dogs are the sentinels of WNV circulation in the Var (12.0%) and Gard (9.1%) departments. All dogs we tested that were positive for IgG were negative for IgM, a finding that indicates infection by the virus did not occur recently.

The results and the statistical analysis reveal notable differences in the seroprevalence rates, according to the geographic area. N’Djamena, Chad, where all native dogs tested positive for WNV, is located at the confluence of the Chari and Logone Rivers and is an area with high densities of residential and migratory birds. In contrast, the northeastern region (Haut-Ogooué) of Gabon, where no native dogs tested positive for WNV, is an ecosystem of wet forests without migratory birds, unfavorable to virus circulation. In Dakar, 18.7% of native dogs were seropositive. In these central parts of Senegal, characterized by a semi-arid climate and vegetation composed of steppe plants and bluegrass, several WNV strains have been isolated from birds and mosquitoes. The seroprevalence was lower (0%–12.5%) in the sub-Saharan area, including Côte d’Ivoire, Gabon, DRC, and Senegal (Siné-Saloum and Casamance), where the humid or semihumid climate is linked with tropical rain forests or woodland savannah known to favor sedentary birds (*5*).

In a large proportion of the human and animal population of Africa, immunity to WNV has developed (*1*). A serologic survey of dogs from the Highveld region of South Africa showed that 37% (138/377) had neutralizing antibodies against WNV (*6*). Similarly, seroprevalence of antibodies against WNV is high among dogs in the United States, for example, 55.9% (218/390) in the Gulf Coast region (*7*). In Turkey, an area where many birds stop over during migration, seroprevalence among dogs was high (37.7%, 43/114) (*8*)*.*

Our study highlights the role of dogs as sentinels for WNV circulation, particularly in southeastern France (Gard and Var departments), where WNV epidemics and epizootics occurred in 2000 and 2003. In addition, we observed that military working dogs purchased from Hungary, where WNV infection is common (*9*), may be seropositive*.* Seroconversion in dogs returning from short missions in WNV-endemic countries such as Chad was also observed. Therefore, our data emphasize the usefulness and convenience of WNV seroprevalence surveys in dogs for studying WNV epidemiology and circulation. It is possible that dogs living close to humans could attract infected mosquitoes, thereby reducing human infection.
